# Direct immunofluorescence-negative pemphigus herpetiformis successfully treated with rituximab

**DOI:** 10.1016/j.jdcr.2026.09.041

**Published:** 2026-09-30

**Authors:** Alexander Gitin, Jacob Kingsley, Joseph S. Durgin, Sara L. Fossum

**Affiliations:** Department of Dermatology, Michigan Medicine, University of Michigan, Ann Arbor, Michigan

**Keywords:** dapsone, direct immunofluorescence, IgA pemphigus, pemphigus herpetiformis, pemphigus, rituximab

## Introduction

Pemphigus herpetiformis is a rare variant of pemphigus characterized by pruritic, annular, and herpetiform eruptions that can clinically resemble dermatitis herpetiformis. Despite its clinical similarity to dermatitis herpetiformis, pemphigus herpetiformis demonstrates immunologic features of pemphigus, most commonly with IgG autoantibodies directed against desmoglein 1 (DSG1). Histopathologic findings are often variable and may lack classic acantholysis, frequently leading to diagnostic delay or misdiagnosis. Direct immunofluorescence (DIF) is typically positive but is not absolutely required for diagnosis.

We report a diagnostically challenging case of pemphigus herpetiformis with negative DIF and refractory disease course, highlighting the importance of integrating clinical, histopathologic, and serologic findings to establish the diagnosis and guide management.

## Case report

A 70-year-old woman was admitted to the hospital for worsening rash. The onset of the rash was 1 year prior, and was associated with pruritus, burning sensation, blistering, and desquamation. Prior treatments, including topical steroids, topical Janus kinase inhibitors, dupilumab, methotrexate 15 mg weekly, and antibiotics (including extended courses of doxycycline 100 mg twice daily), proved unsuccessful. Dapsone had precipitated methemoglobinemia at a dose of 100 mg daily but was not effective at lower doses. Systemic steroids led to temporary improvement, with rebounding after discontinuation. She was not treated with mycophenolate mofetil.

Physical examination revealed erythematous, scaly plaques on the scalp, forehead, eyelids, and cheeks. On the chest and back were erythematous annular and polycyclic plaques with scattered papules, pustules and vesicles (Fig 1, *A* and *B*).

**Fig 1:**
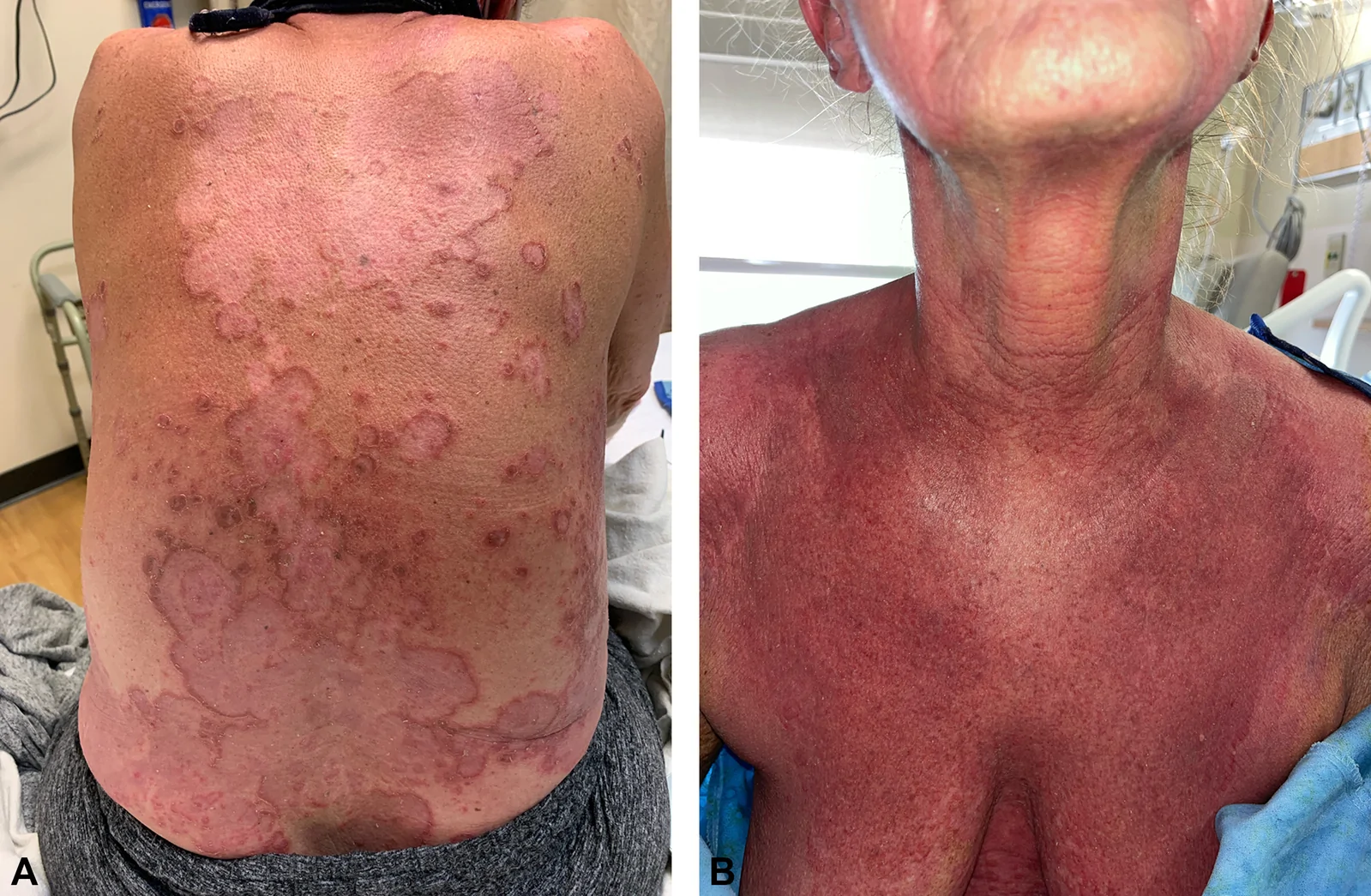
**A** and **B,** Clinical presentation consisting of erythematous, scaly plaques, some in an annular or polycyclic arrangement, with scattered papules, pustules and vesicles.

The patient had undergone 3 previous punch biopsies, which revealed somewhat variable findings, including subacute spongiotic dermatitis, impetiginized spongiotic dermatitis with superficial lymphoplasmacytic infiltrate and rare eosinophils, and subcorneal pustular dermatitis with variable background neutrophilic spongiosis. A repeat punch biopsy obtained during admission showed subcorneal and intracorneal neutrophilic inflammation with spongiosis, focal acantholysis and rare eosinophils (Fig 2). Grocott Methenamine stain was negative for fungal organisms. DIF was negative for immune deposition on 3 separate occasions.

**Fig 2:**
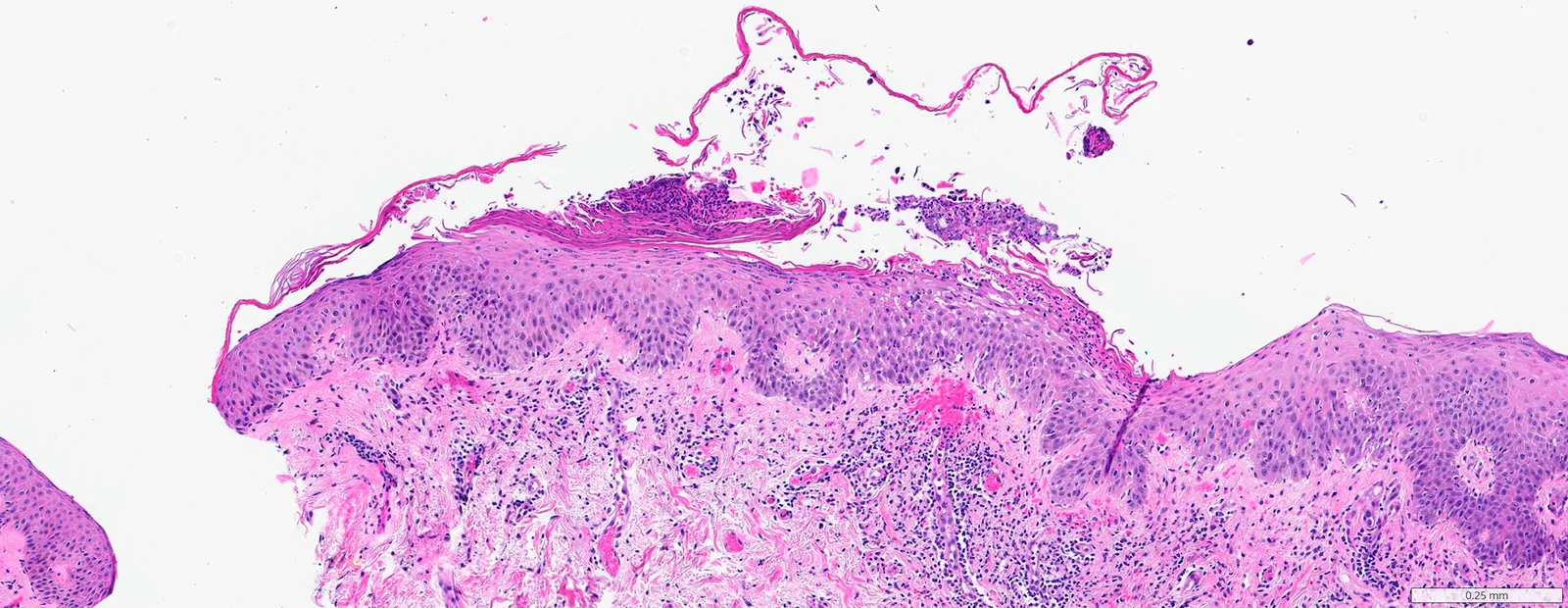
Histopathology showing subcorneal and intracorneal neutrophilic inflammation with spongiosis, focal acantholysis and rare eosinophils.

Previous evaluation also included a negative antinuclear antibody screen and negative Jo-1 and smooth muscle antibodies. Zinc and vitamin B12 levels were within normal limits. Serum protein electrophoresis and immunofixation demonstrated a mildly decreased IgG. Serum enzyme-linked immunosorbent assay for desmogleins revealed IgG autoantibodies against DSG1 of over 200 RU/mL with no elevation in desmoglein 3 autoantibodies. Indirect immunofluorescence was positive for epithelial cell surface IgG and IgG4 antibodies and negative for circulating IgA antibodies against the basement membrane and epithelial cell surface.

The patient was discharged on a prednisone taper and subsequently seen in the outpatient setting, at which time colchicine was initiated in the setting of active infection. Colchicine was discontinued due to side effects and lack of efficacy. The patient then received 2 doses of rituximab 1000 mg, administered 2 weeks apart. At follow up 2 weeks after her first dose of rituximab, her rash had completely resolved while on 0.8 mg/kg of prednisone (Fig 3). Her prednisone was subsequently tapered off over 4 months without flaring of her disease. She underwent a second cycle of rituximab 1000 mg, 2 doses, administered 2 weeks apart and 6 months following her first cycle. She remained disease-free at follow-up 7 months after her first rituximab cycle and 2 months after completing her prednisone taper.

**Fig 3:**
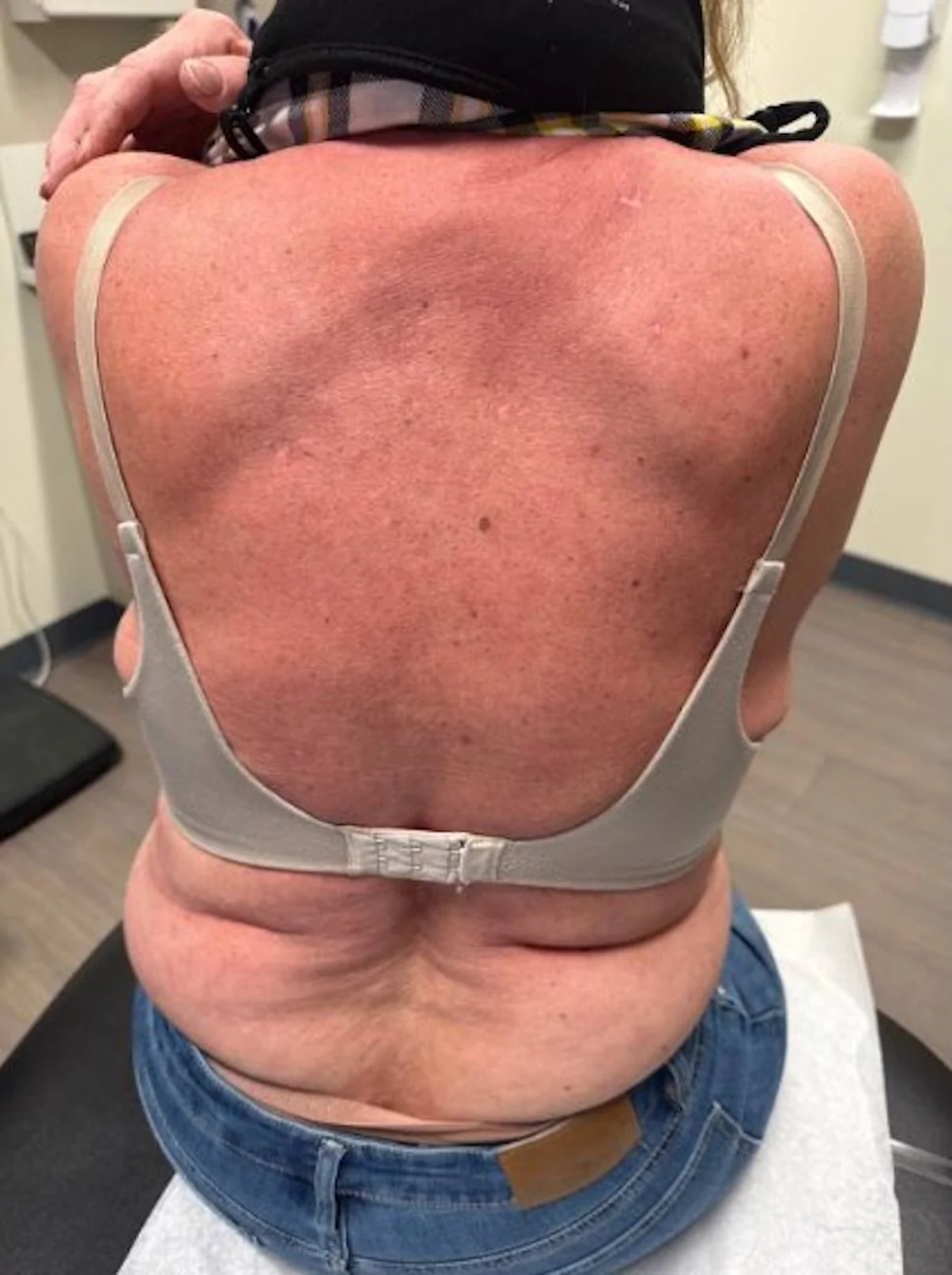
Resolution of her cutaneous disease 2 weeks after her first dose of rituximab.

## Discussion

Pemphigus herpetiformis is a rare autoimmune blistering disease first described by Jabłońska and colleagues in 1975.^1^ It accounts for approximately 6% to 7% of all pemphigus cases. There does not appear to be a clear sex or ethnic predominance. Clinical presentation is characterized by erythematous, vesiculobullous and papular lesions, often arranged in a grouped or annular distribution.^2^ It has rarely been associated with malignancy, HIV infection, autoimmune hemolytic anemia, psoriasis, and systemic lupus erythematosus.^2^

Histopathology is distinctive compared to other forms of pemphigus, as acantholysis is not always present. Spongiosis and intraepidermal abscesses, composed of neutrophils and/or eosinophils, are characteristic. DIF is positive for intercellular IgG and C3 deposition in most cases, however, this is not absolutely required for diagnosis.^2^, ^3^ In the case of this patient, DIF was negative on 3 separate biopsies, complicating the diagnosis. Indirect immunofluorescence typically shows intercellular IgG binding on epithelial substrates, similar to other pemphigus variants. Enzyme-linked immunosorbent assay reactivity against DSG1, and less often desmoglein 3, is characteristic.^2^ Because of the variable histopathologic findings, diagnosis can be challenging.

The differential diagnosis of pemphigus herpetiformis primarily includes dermatitis herpetiformis as well as other disorders within the pemphigus group (including IgA pemphigus and pemphigus erythematosus), linear IgA bullous dermatosis, and subcorneal pustular dermatosis. Diagnostic criteria have been proposed by Costa et al, requiring 1 clinical, 1 pathologic, and 1 immunologic criterion.^3^ In this case, our patient met clinical criteria through the presence of pruritic annular plaques and herpetiform blisters, pathologic criteria through the presence of both intraepidermal eosinophils and neutrophils, and immunologic criteria through detection of IgG serum antibodies against DSG1. Distinguishing IgA pemphigus from pemphigus herpetiformis can prove especially challenging due to overlapping clinical and histopathologic features, including circinate vesiculopustular eruptions and often neutrophil-predominant infiltrates with subcorneal pustules.^2^, ^4^

Systemic corticosteroids are the mainstay of treatment for pemphigus herpetiformis. Sulfonamides such as dapsone, and sulfasalazine have been helpful in prior reports.^2^, ^5^ Compared with other types of pemphigus, pemphigus herpetiformis follows a more indolent course, and response to treatment with dapsone or low dose systemic corticosteroids is typically excellent.^2^ In refractory cases, traditional immunosuppressants may be considered. Several recent case reports have demonstrated successful treatment with rituximab, however, consensus regarding an optimal regimen has not been established.^6^, ^7^, ^8^, ^9^

This challenging case highlights important points. It is essential to keep pemphigus herpetiformis on the differential diagnosis for rashes that are annular, herpetiform or vesiculobullous, especially with its variable histology. Even in the setting of a negative DIF, if a rash is clinically or histologically concerning for pemphigus herpetiformis, obtaining indirect immunofluorescence and/or serologies for DSG1 and desmoglein 3 may lead the clinician to this diagnosis. Future studies are warranted to investigate the frequency of occurrence of DIF-negativity amongst pemphigus herpetiformis patients. This case also redemonstrates that rituximab is a treatment option for patients who do not respond to or cannot tolerate first-line therapies such as dapsone or oral steroids.

### Declaration of generative AI and AI-assisted technologies in the writing process

None.

## Conflicts of interest

None disclosed.
